# C-Reactive Protein Level as a Novel Serum Biomarker in Sarcopenia

**DOI:** 10.1155/2024/3362336

**Published:** 2024-08-20

**Authors:** Shangjin Lin, Xiuxiu Chen, Ying Cheng, Hou Huang, Fengjian Yang, Zhijun Bao, Yongqian Fan

**Affiliations:** ^1^ Department of Orthopedics Huadong Hospital Affiliated to Fudan University, Shanghai 200040, China; ^2^ Shanghai Key Laboratory of Clinical Geriatric Medicine, Shanghai 200040, China; ^3^ Department of Gerontology Huadong Hospital Affiliated to Fudan University, Shanghai 200040, China; ^4^ Department of Gastroenterology Huadong Hospital Affiliated to Fudan University, Shanghai 200040, China

## Abstract

**Background:**

The role of C-reactive protein (CRP), an inflammatory marker, in the development of sarcopenia remains uncertain.

**Methods:**

This cross-sectional research involved the enrollment of 207 patients, classified into two groups: 74 patients with sarcopenia and 133 patients without sarcopenia. Clinical data of the participants, including hand grip strength, walking speed, appendicular lean mass (ALM), and calf circumference, were collected and recorded. We evaluated the extent to which CRP levels are associated with the risk of sarcopenia using both univariate and multivariate logistic regression models. Besides, the correlation between CRP levels, hand grip strength, ALM, and walking speed was examined using the Spearman rank correlation test. Moreover, we have employed the Mendelian randomization (MR) analysis technique to explore the causal relationship between CRP levels and the occurrence of sarcopenia.

**Results:**

The sarcopenia group showed a higher proportion of older women, with significant differences in anemia prevalence, calf circumference, gait speed, ALM, hand grip strength, and elevated CRP levels compared to the control group. Logistic regression analyses identified CRP as an independent risk factor for sarcopenia (OR: 1.151, 95% CI:1.070−1.238, and *P* < 0.001). Correlation analysis results revealed a noteworthy inverse association with hand grip strength (*R* = −0.454 and *P* < 0.001), ALM (*R* = −0.426 and *P* < 0.001), and walking speed (*R* = −0.431 and *P* < 0.001). MR analysis provided further evidence of a significant detrimental link between genetically predicted CRP levels and essential sarcopenia characteristics, with consistent results across various statistical models.

**Conclusions:**

Our study uncovered strong evidence supporting a noteworthy inverse association and causality between CRP concentrations and sarcopenia, indicating that CRP has the potential to serve as a biomarker for sarcopenia.

## 1. Introduction

Sarcopenia, a chronic disease that often occurs with age, is marked by a progressive decrease in muscle mass, strength, and function [1]. This condition, primarily affecting older adults, is prevalent and has a considerable impact on overall health and quality of life, leading to falls, fractures, and other injuries [2]. The prevalence of sarcopenia is influenced by factors such as the population being studied and the diagnostic criteria being used. Various organizations and researchers have suggested different criteria for diagnosing sarcopenia, resulting in variations in prevalence estimates. In the case of older Asian individuals, the prevalence of sarcopenia ranges from 5.5% to 25.7%, based on the diagnostic criteria established by the Asian Working Group for Sarcopenia (AWGS) [3]. According to the definition provided by the European Working Group on Sarcopenia in Older Adults (EWGSOP), the occurrence rate of sarcopenia among elderly individuals residing in the community is 5.1% for males and 11.8% for females [4]. Additionally, the prevalence of sarcopenia tends to increase with age.

The process of sarcopenia's pathogenesis is intricate and multifaceted. It encompasses age-related changes, lifestyle factors, and biological processes that lead to the decline in muscle mass, strength, and function. Inflammaging, a persistent form of low-grade inflammation, is thought to play a crucial role in the development of sarcopenia [5]. Inflammaging is commonly observed in aging individuals and can have detrimental effects on skeletal muscle health. C-reactive protein (CRP) is frequently employed as a biomarker to assess systemic inflammation, which is closely related to age-related degenerative changes [6]. Hence, increased levels of CRP may reflect an inflammatory condition linked to muscle atrophy and compromised muscle performance. Nevertheless, the investigations exploring the correlation between CRP concentrations and sarcopenia yield diverse findings. This variation could stem from several factors, including limitations in sample sizes, divergences in study designs, and the presence of potential confounding variables. Considering these factors, our study was designed to comprehensively investigate the association between CRP levels and sarcopenia. We initiated a case–control study by enrolling 74 sarcopenia patients and 133 nonsarcopenia patients, aiming to evaluate the significance of CRP as a stand-alone predictor for sarcopenia. Furthermore, to bolster our findings with more robust evidence, we employed Mendelian randomization (MR) analysis, utilizing genetic variations in CRP as the exposure variable, to investigate the prospective causal association between CRP and sarcopenia.

MR analysis is a statistical technique that utilizes genetic variants or single nucleotide polymorphisms (SNPs) as instrumental variables (IVs) to investigate causal relationships between risk factors (exposure) and outcomes (disease or trait), minimizing confounding bias and addressing issues of reverse causation [7]. To date, there is a lack of academic literature exploring the correlation between CRP and sarcopenia through the MR method.

## 2. Materials and Methods

### 2.1. Research Design

The purpose of this cross-sectional investigation was to examine the correlation between levels of CRP in the serum and the occurrence of sarcopenia. The study assessed the role of serum CRP as an independent predictor of sarcopenia by systematically collecting and analyzing the clinical data, blood specimens, and performing quantitative assessments of muscle function among participants. Additionally, we employed MR analysis to examine the possible causal relationship between genetic variations in CRP and sarcopenia. The MR analysis in the study consisted of four main steps: (1) identification of IVs significantly associated with CRP, (2) assessment of the relationship between the selected IVs and sarcopenia, (3) estimation of the causal effect between CRP and sarcopenia, and (4) evaluation of the MR results through pleiotropy and sensitivity analyses.

### 2.2. Study Participants

Between October 2022 and October 2023, a cross-sectional investigation was carried out at Huadong Hospital affiliated to Fudan University. A total of 207 participants, all aged over 40, were selected to form the cohort. This cohort included 74 sarcopenia patients and 133 nonsarcopenic controls. These criteria ruled out individuals with significant cardiovascular, renal, or hepatic conditions; those who had undergone major surgery or experienced severe trauma recently; patients with neuromuscular or rheumatic diseases; and those on long-term medication regimes potentially impacting muscle metabolism. Prior to their involvement in the study, informed consent was duly obtained from all participants after the study protocol received approval from the Ethics Committee of Huadong Hospital affiliated to Fudan University (2023K201).

### 2.3. Clinical Data Collection

Detailed clinical information, encompassing age, gender, body mass index (BMI), and calf circumference, was systematically gathered from all subjects. To ensure accuracy, calf circumference was measured on both sides, with the greater value recorded for analysis. Additionally, blood samples were obtained from all participants to determine serum CRP levels and complete blood cell counts. The blood tests were conducted the following morning after a minimum fasting period of 8 hr, with samples collected from a peripheral vein in the forearm.

### 2.4. Diagnosis of Sarcopenia

Under the AWGS diagnostic guidelines [3], sarcopenia is identified as an inadequate appendicular lean mass (ALM) accompanied by a minimum of one of the subsequent circumstances, low muscle strength or low physical performance.

### 2.5. Determination of ALM

The study assessed the measurement of ALM through bioelectric impedance analysis (BIA). BIA is widely recognized as a noninvasive and relatively straightforward technique for estimating ALM [8]. This method operates by transmitting a low-intensity electrical current through the body and quantifying the resultant impedance variances among different tissues, such as muscle and fat. The estimation of ALM is derived from these variations in impedance. As per the 2019 consensus established by the AWGS [3], males with muscle mass below 7.0 kg/m^2^ and females with muscle mass below 5.7 kg/m^2^ are categorized as having low muscle mass.

### 2.6. Determination of Skeletal Muscle Function

This research aimed to evaluate muscle strength through the utilization of a Jamar dynamometer to quantify grip strength, along with a 6-m walking test to evaluate physical performance. The grip strength test involved participants sitting with their shoulder joint slightly abducted, and their arm and forearm forming a 90° angle, while exerting maximum force on the dynamometer. The highest value obtained from both hands was recorded as the indicator of muscle strength. Based on the AWGS standards, individuals were categorized as having low muscle strength if their grip strength was below 28 kg for males and 18 kg for females [3]. For the walking test, a 10 m straight path was marked with intervals at the start, 3 m, 9 m, and end. Participants started walking from the starting point and were timed between the 3 and 9 m marks to calculate their walking speed. According to the AWGS guidelines, the criteria for determining low physical performance was the presence of a walking speed below 1.0 m/s over a distance of 6 m [3].

### 2.7. Data Sources for Exposure

To more reliably discern the correlation between CRP and sarcopenia, our study utilized Genome-Wide Association Study (GWAS) data from a large cohort, encompassing 436,939 individuals [9]. The CRP level data from the GWAS summary statistics (Table 1) was acquired from the IEU GWAS database (https://gwas.mrcieu.ac.uk/datasets/). In order to guarantee the precision of the findings obtained from MR analysis, an intentional effort was made to limit the impact of population stratification factors by exclusively enrolling individuals with European descent as participants in this investigation.

### 2.8. Data Sources for Outcome

Due to the recent establishment of the definition and diagnostic criteria for sarcopenia, we were unable to find the relevant information on sarcopenia in IEU GWAS database. As a result, the sarcopenia-related traits (hand grip strength, walking space, and ALM) were selected as the outcome measures for estimating MR in this study. These traits are commonly used in clinical diagnosis of sarcopenia. Hand grip strength is commonly acknowledged as an effective measure of muscle strength and overall muscle function [10]. The United Kingdom Biobank (UKB) provided the summary statistic data for hand grip strength in the GWAS. The data included information from 335,842 individuals for right-hand grip strength and 335,821 individuals for left-hand grip strength. The GWAS ID for right-hand grip strength is ukb-a-374, while the GWAS ID for left-hand grip strength is ukb-a-379. ALM, an integral part of multiple diagnostic criteria for sarcopenia, denotes the mass of skeletal muscles in the limbs, as underscored by criteria from both the EWGSOP and the Foundation for the AWGS [2, 3]. The statistical data summary of the ALM was acquired from a GWAS that comprised a grand total of 244,730 participants (GWAS ID: ebi-a-GCST90000027) within a discovery cohort [11]. This cohort study utilized BIA to measure the combined mass of the limbs to calculate the ALM data. The standardized genetic values of ALM were adjusted for age, sex, appendicular fat mass, and other principal components, and were corrected accordingly. Walking pace, a key indicator of physical performance and functional mobility, is notably impacted by sarcopenia. A reduced gait speed is a significant marker, reflecting the muscle function deterioration associated with sarcopenia [12]. To gather information on walking pace, we obtained summary statistics from the GWAS database, which comprised data from 335,349 individuals (GWAS ID: ukb-a-513). To mitigate the potential influence of population stratification, all enrolled individuals were exclusively of European ancestry. Detailed information about the cohorts of outcomes can be found in Table 1.

### 2.9. Selection of Instrumental Variables

**Figure 1:**
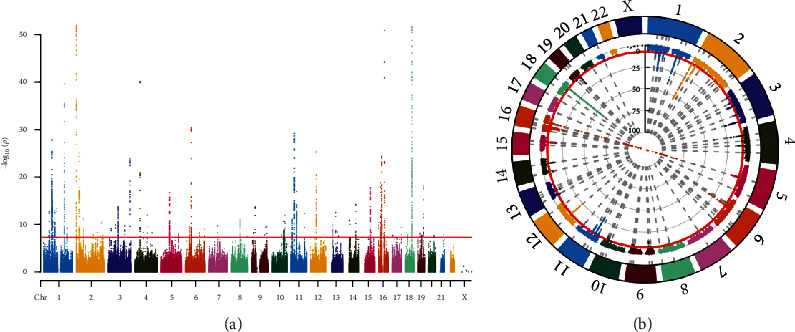
Rectangular-Manhattan plot (a) and circular-Manhattan plot (b) of GWAS results on chromosome with significant association with CRP (*p* < 5 × 10^−8^).

To obtain instrumental SNPs that meet the requirements, we performed a series of quality control steps on the GWAS summary data of CRP level. To ensure unbiased results, it is imperative for the chosen IVs to adhere to three fundamental assumptions of MR analysis [13]: (1) the selected IVs must demonstrate a connection with the targeted exposure; (2) the IVs must not exhibit any correlation with potential confounding factors that might sway the outcome variable; and (3) the IVs should solely affect the outcome by means of their impact on the exposure, and not through other pathways or pleiotropy. As depicted in Figure 1, the initial validation of the first hypothesis in the MR analysis involved identifying genome wide SNPs that demonstrated a notable correlation with CRP (*p* < 5 × 10^−8^). Besides, confirming the absence of linkage disequilibrium (LD) is vital when assessing the selected instrumental SNPs for analysis. The presence of strong LD among the instrumental SNPs may introduce potential bias, consequently jeopardizing the credibility of the results. Hence, to secure precise and dependable conclusions, it is imperative to guarantee LD absence for the instrumental SNPs under examination. The clumping process was carried out on the European samples obtained from the 1,000 genomes project data, using a window size of 10,000 kb and a significance threshold of *R*^2^ < 0.001 [14]. Next, we calculated the *F* values of all SNPs to test whether the selected IVs were weakly correlated with exposure. The formula for calculating the F-statistic can be expressed as follows:

(1) $$\begin{array}{c} F=\frac{R^{2}\left(n-k-1\right)}{k\left(1-R^{2}\right)} \end{array}$$

where the sample size of the CRP level GWAS study is denoted by n, the number of IVs is represented by k, and R^2^ indicates the extent to which the selected IVs account for the variation in exposure [15]. If the F-statistic exceeds a value of 10 for the association between the IVs and exposure, it is highly improbable that weak instrumental variables are present [16]. Next, the necessary information about each instrumental variable obtained from the GWAS summary data of CRP level was investigated using the PhenoScanner database. The required data was gathered by researchers who accessed http://www.phenoscanner.medschl.cam.ac.uk/. This was done to assess whether the selected IVs met the requirements outlined in principal assumptions (2) and (3). Subsequently, we conducted a thorough search to locate the specified IVs using GWAS summary statistics of traits associated with sarcopenia.

### 2.10. MR Analysis

For this research, a MR analysis was performed using CRP level as the exposure variable and emphasizing traits associated with sarcopenia as the outcome variable. To address the potential impact of genetic diversity or measurement inaccuracies in population data on the outcomes of MR, we employed the MR pleiotropy residual sum and outlier (MR-PRESSO) approach to detect and eradicate pleiotropic IVs [17]. We utilized the MR-PRESSO technique to detect and rectify anomalies in the linear regression of inverse-variance weighted (IVW), which served as the primary approach for conducting MR analysis. In this study, a distribution number of 3,000 was set for the MR-PRESSO analysis. Subsequently, we proceeded with the MR analysis by removing pleiotropic outlier IVs with the assistance of MR-PRESSO. Five different methods were used to ascertain the causal link between sarcopenia and CRP level. These methods included IVW, Mendelian randomization-Egger (MR-Egger), weighted mode, weighted median, and simple mode. The most reliable method for conducting MR analysis among these approaches is recognized as the IVW method. Using a meta-analysis approach of IVW, the IVW technique integrates the Wald estimates of each IV in order to provide a comprehensive evaluation of the impact of CRP level on sarcopenia [18]. We eliminated the need for Ethics Committee approval by utilizing publicly accessible GWAS summary data.

### 2.11. Sensitivity and Heterogeneity Analysis

To ensure the reliability and robustness of our MR findings, various techniques were implemented in this research. To commence, Cochran's Q statistic was employed to examine heterogeneity in the data, with a significance threshold set at *P* value < 0.05. Moreover, funnel plots were utilized for visual representation to detect any potential heterogeneity. After analyzing the outcomes of the Cochran's Q test, a decision was made to select either the fixed-effects model of IVW or the random-effects model of IVW. The MR-PRESSO technique focuses on detecting and rectifying potential anomalies in the genetic association data and assess the presence of heterogeneous causal estimates due to pleiotropy. Besides, the MR-Egger intercept examination serves as a valuable mechanism to identify potential horizontal pleiotropy within instruments [19]. The analysis of leave-one-out was performed to assess influential variants, detect potential biases or violations of MR assumptions, and provide insights into the overall robustness of the causal estimates. By employing the methods described above, we strived to enhance the credibility and dependability of our MR results in establishing a potential link between CRP concentration and sarcopenia.

### 2.12. Statistical Analysis

The presentation of categorical variables consisted of counts and percentages, and the *χ*^2^ test was utilized to assess group differences. To evaluate the normality of continuous variables, the Kolmogorov–Smirnov test was employed. For normally distributed variables, means ± standard deviations were used for expression, and Student's *t*-test was applied for group comparisons. Intergroup comparisons were conducted using the Mann–Whitney *U* test to describe nonnormally distributed variables, represented by medians and interquartile ranges (IQR). Univariate and multivariate binary logistic regression analyses were undertaken to examine the correlation between CRP and sarcopenia. The statistical calculations were executed by utilizing SPSS software (version 25.0, SPSS Inc., Chicago, USA). R version 4.1.3 and the R packages two-sample MR and MR-PRESSO were utilized for conducting all MR analyses. The outcomes of the MR analysis displayed the odds ratio (OR) along with the 95% confidence interval (CI) to assess the relative risk associated with CRP-induced sarcopenia. To indicate statistical significance, a *P* value below 0.05 was considered.

## 3. Results

### 3.1. Comparison of Demographic and Clinical Characteristics

**Figure 2:**
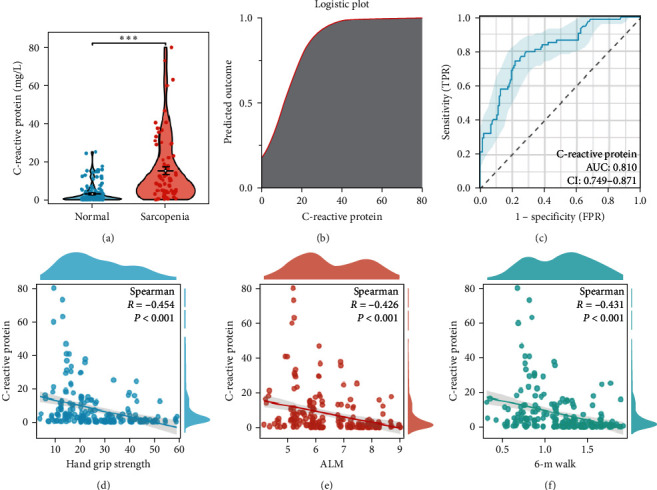
The association between CRP and sarcopenia. (a) Violin plot of the difference in CRP levels between the sarcopenia and nonsarcopenia groups. (b) Logistic plot from binary logistics regression of CRP predicting the occurrence of sarcopenia. (c) The ROC curve of CRP for predicting sarcopenia. (d) Scatterplot of correlation between CRP and hand grip strength. (e) Scatterplot of correlation between CRP and ALM. (f) Scatterplot of correlation between CRP and 6-m walk.  ^*∗∗∗*^indicates a *P*-value of less than 0.001, denoting a statistically significant difference.

Of the 207 subjects meeting the inclusion criteria, 74 (35.75%) were diagnosed with sarcopenia. In the sarcopenia group, a notable majority of 71.62% were women, significantly higher than the 42.11% in the control group. The median age of those with sarcopenia was 75 years (IQR: 69–80 years), markedly older than the control group's median age of 59 years (IQR: 50–67 years). Furthermore, the occurrence of anemia exhibited a notably higher incidence among individuals in the sarcopenia category, amounting to 13.51%. This proportion is in stark contrast to the mere 1.5% among participants in the control group. Consistent with the expectations, patients with sarcopenia exhibited significantly reduced calf circumference (30.4 (27.9, 32.1) cm vs. 34.1 (32.3, 36.0) cm; *P* < 0.001), slower gait speed (0.78 ± 0.19 m/s vs. 1.39 ± 0.25 m/s; *P* < 0.001), lower ALM (5.25 (5.11, 5.84) kg/m^2^ vs. 7.32 (6.14, 7.98) kg/m^2^; *P* < 0.001), and decreased hand grip strength (14.4 (11.4, 16.5) kg vs. 32.2 (23.8, 42.1) kg; *P* < 0.001). Moreover, the sarcopenia group exhibited considerably increased serum CRP levels (8.94 (3.92, 21.64) mg/L compared to 1.55 (0.67, 4.01) mg/L; *P* < 0.001), indicating a statistical significance, as illustrated in Figure 2(a). Remarkably, the count of leukocytes exhibited a significant decline in the group with sarcopenia in comparison to the nonsarcopenia group (*P*=0.024). Surprisingly, despite the increase in neutrophil count, no statistically meaningful distinction was observed (*P*=0.294). The fundamental features of the participants, distinguishing between individuals with and without sarcopenia, can be found in Table 2.

### 3.2. The Relationship between CRP and Sarcopenia

Table 3 demonstrates that the univariate binary logistic regression model discovered a substantial inverse association between sarcopenia and serum CRP concentrations (OR: 1.152, 95% CI:1.094−1.214, and *P* < 0.001). According to the probability plot of logistic regression (Figure 2(b)), it can be observed that the risk of sarcopenia increases in proportion to the elevation in CRP levels. Additionally, the high diagnostic efficacy of CRP in identifying sarcopenia is further confirmed by the receiver operating characteristic (ROC) curve shown in Figure 2(c). The area under the curve (AUC) value, which is 0.81 (95% CI: 0.749−0.871), indicates the accuracy of the CRP test in diagnosing sarcopenia. Through adjustment for various potential confounders, including age, gender, anemia, and leukocyte count, multivariate binary regression analysis was performed to further validate the status of CRP as an autonomous risk factor for sarcopenia (OR: 1.151, 95% CI: 1.070−1.238, and *P* < 0.001). Besides, to elucidate the relationship between serum CRP levels and sarcopenia-related traits, we employed Spearman rank correlation test. This analysis demonstrated significant negative correlations between serum CRP levels and ALM and hand grip strength and 6-m walking speed (Figures 2(d), 2(e), and 2(f)).

### 3.3. Filtering of SNPs

Our study aimed to investigate the causal link between CRP and sarcopenia. In order to accomplish this objective, we initially acquired 252 LD-independent SNPs (with *R*^2^ values below 0.001 and a window size of 10,000 kb), which exhibited a robust correlation with CRP (*P* < 5 × 10^−8^). By employing the MR-PRESSO technique to eliminate SNPs that exhibit pleiotropic effects, we successfully identified 237, 233, 207, and 237 SNPs as IVs in the MR analysis conducted for left-grip strength, right-grip strength, ALM, and walking pace, correspondingly.

### 3.4. Causality of Genetically Predicted CRP on Hand Grip Strength

**Figure 3:**
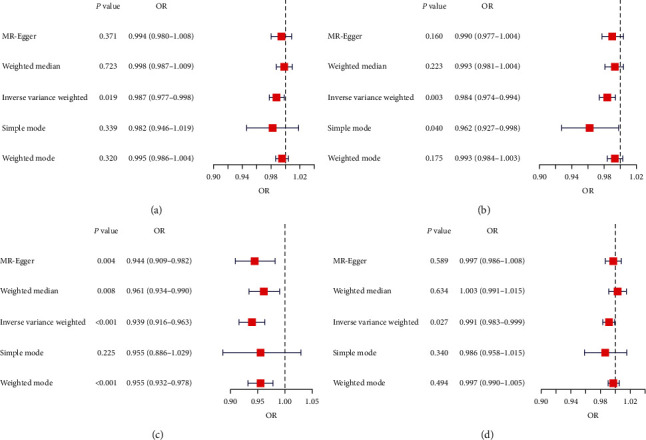
Forest plots of MR results from different methods for assessing the causal effect of CRP on sarcopenia-related traits. (a) CRP-grip strength (left). (b) CRP-grip strength (right). (c) CRP–ALM. (d) CRP–walking pace.

**Figure 4:**
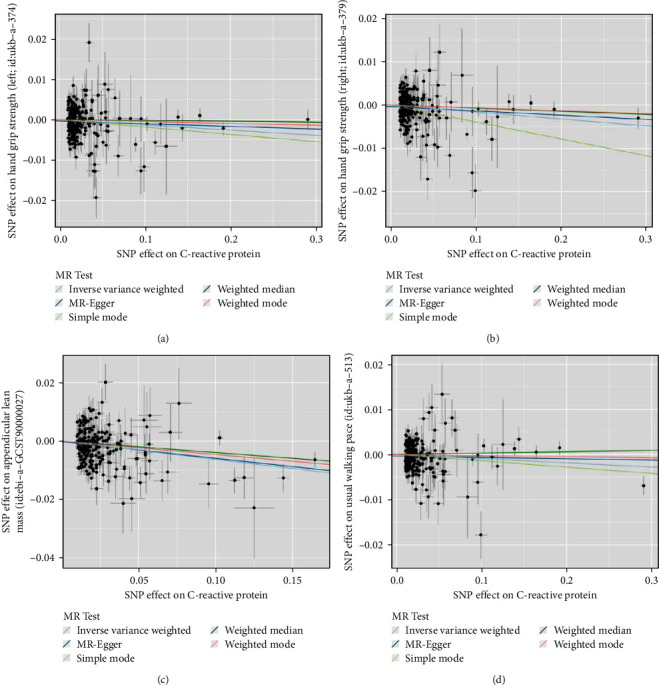
Scatter plots for MR analyses. (a) CRP-grip strength (left). (b) CRP-grip strength (right). (c) CRP–ALM. (d) CRP–walking pace.

**Figure 5:**
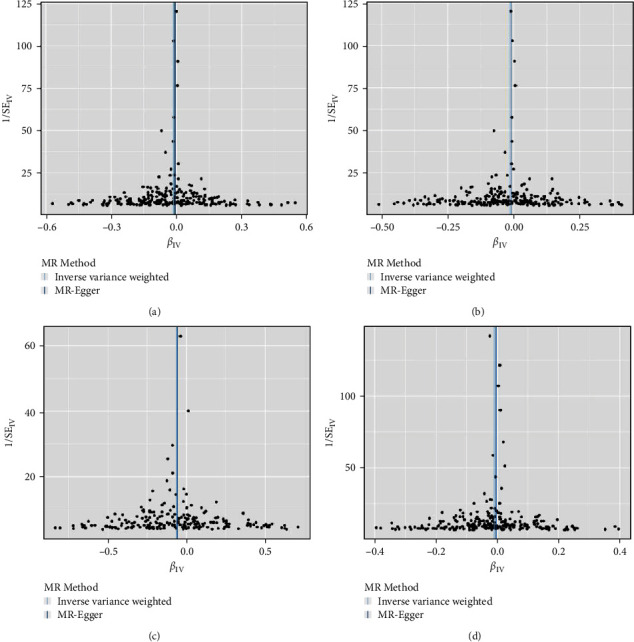
Funnel plots for MR analyses. (a) CRP-grip strength (left). (b) CRP-grip strength (right). (c) CRP–ALM. (d) CRP–walking pace.

Based on the findings from the MR analysis (Figures 3(a) and 4(a)), it was established that a noteworthy inverse causal association existed between CRP concentrations and left-hand grip strength (OR: 0.987, 95% CI: 0.977–0.998, and *P*=0.019), as assessed using the IVW-random effects method. The regression analysis conducted by MR-Egger did not uncover any indication of directional pleiotropy among SNPs linked to CRP (intercept = −0.0004 and *P*=0.17). Likewise, there is significant evidence establishing a causal link between genetically projected CRP levels and right-hand grip strength (OR: 0.939, 95% CI: 0.916–0.963, and *P*=0.003; Figures 3(b) and 4(b)). The main role of the funnel plot in MR analysis is to evaluate whether there is asymmetry in the distribution of study estimates. As shown in Figures 5(a) and 5(b), the funnel plot revealed no evidence of asymmetry. In the forest plot (Figures S1(a) and S1(b)), we can observe the impact of each SNP on the susceptibility to hand grip strength. The employment of the leave-one-out methodology in our analysis reveals that the stability of the MR results is preserved, and the presence of any specific SNP does not exert any influence on the outcomes (Figures S2(a) and S2(b)).

### 3.5. Causality of Genetically Predicted CRP on ALM

Notable variations among these 207 SNPs were identified by conducting the Cochran's Q test (*P* < 0.05). Consequently, the random-effects model was applied by the IVW method during MR analysis. According to the findings from the regression analysis conducted by MR-Egger, there was no indication of directional pleiotropy observed among the SNPs examined (intercept = −0.0002 and *P*=0.698), as shown by the funnel plot (Figure 5(c)). The IVW-MR analysis yielded noteworthy evidence of a detrimental causal link between genetically anticipated CRP and ALM (OR: 0.939, 95% CI: 0.916–0.963, and *P* < 0.001; Figure 3(c)). The scatter plot in Figure 4(c) illustrates a discernible decline in ALM corresponding to the increase in genetically predicted CRP levels. Additionally, findings from both the weighted median method (OR: 0.961, 95% CI: 0.934–0.990, and *P*=0.008; Figure 3(c)) and the weighted mode method (OR: 0.955, 95% CI: 0.932–0.978, and *P* < 0.001; Figure 3(c)) corroborated the results of the IVW method. These alternative methods provided consistent results, further validating the conclusions drawn from the IVW analysis. The effect of each SNP on ALM can be demonstrated through the forest plot (Figure S1(c)). The analysis of leaving-one-out (Figure S2(c)) verifies the consistency of the results obtained from the IVW-MR analysis, suggesting the absence of any significant impact from any specific SNP.

### 3.6. Causality of Genetically Predicted CRP on Walking Pace

To address the heterogeneity among SNPs, a random effects model was incorporated into the IVW-MR analysis, which revealed significant variations (*P* < 0.001). Furthermore, our IVW-MR analysis identified an inverse causal link between CRP levels and walking pace (OR: 0.991, 95% CI: 0.983–0.999, and *p*=0.027; Figures 3(d) and 4(d)). The forest plot (Figure S1(d)) visually details the causal effects of individual SNPs on walking pace. Furthermore, the analysis of leaving-one-out (Figure S2(d)) provides additional evidence regarding the reliability of the IVW-MR analysis outcomes, illustrating that the outcomes are not significantly influenced by any individual SNP.

## 4. Discussion

Sarcopenia is a multifactorial condition influenced by various factors such as age, hormonal changes, physical inactivity, poor nutrition, and chronic diseases [20, 21, 22]. Early detection and intervention are crucial in managing sarcopenia and preserving muscle mass and function. Inflammation can be triggered by various factors, including age-related changes, oxidative stress, sedentary lifestyle, obesity, and chronic diseases [23]. Elevated levels of various inflammatory biomarkers, such as CRP, IL-6, and TNF-*α*, have been associated with greater muscle depletion, decreased muscle power, and functional deterioration among elderly individuals [24]. CRP, as an inflammatory biomarker, can indicate the presence of underlying systemic inflammation that may accelerate the progression of sarcopenia. Despite this, the correlation between CRP levels and sarcopenia remains to be conclusively determined. In order to develop early detection and intervention for sarcopenia, it is essential to establish a definitive correlation between CRP levels and sarcopenia risk. Consequently, our study focused on examining the link between CRP concentrations and sarcopenia.

The onset of sarcopenia is strongly linked to elevated CRP levels, as revealed by our research findings. Furthermore, it is worth noting that this connection remains evident even after accounting for various factors that could potentially influence the results, such as age, gender, anemia, and leukocyte count. These findings suggest that CRP might have potential as a biomarker for predicting the onset of sarcopenia. The validity of CRP as a sarcopenia predictor was further corroborated by ROC curve analysis. Additionally, our correlation analysis revealed a substantial inverse association linking the levels of CRP in serum and critical factors associated with sarcopenia, including strength of grip, ALM, and velocity while walking a distance of 6 m. These insights underscore the potential value of CRP as a means of assessing individuals in clinical practice. To explore the potential causal connection between CRP levels and three significant sarcopenia-related traits, this research utilized the two-sample MR approach. By considering the temporal sequence of causality and accounting for confounding factors, we aimed to overcome the inherent limitations posed by cross-sectional studies.

The diagnosis of sarcopenia heavily relies on hand grip strength, given its significant association with overall muscle strength, predictive value for adverse health outcomes, inclusion in diagnostic criteria, and ability to monitor treatment efficacy [25]. According to a longitudinal investigation, a reverse relationship has been found between increased levels of CRP and the strength of hand grip [26]. In another cross-sectional study conducted in Korea, 2,171 postmenopausal women aged 45 years and above were recruited [27]. This study also observed a negative and independent association between serum levels of CRP and relative hand grip strength. Our own cross-sectional study produced similar results, demonstrating a significant negative correlation between CRP levels and hand grip strength (*R* = −0.454 and *P* < 0.001; Figure 2(d)). Additionally, the outcomes derived from MR analysis have also uncovered a substantial adverse causal correlation amid levels of CRP and hand grip strength, exerting an impact on both the dexterous and sinistral hand. This observation implies that elevated CRP levels could be indicative of decreased muscle function, particularly in terms of reduced grip strength.

In a 2010 published consensus, the EWGSOP suggested that sarcopenia can be diagnosed based on the presence of insufficient muscle mass [28]. Previous investigations have uncovered that heightened levels of CRP are connected with decreased muscle mass. A comprehensive analysis of 17 investigations encompassing 11,249 subjects (3,072 diagnosed with sarcopenia and 8,177 as controls) revealed that individuals affected by sarcopenia exhibited decidedly elevated CRP levels in comparison to their nonsarcopenic counterparts [29]. It was worth noting that the detection of sarcopenia in this meta-analysis primarily relied on muscle mass indicators. In a different meta-analysis conducted with a considerable sample size of 76,889 individuals, a notable inverse association (*R* = −0.12 and *p* < 0.001) was identified between elevated CRP levels and muscle mass [24]. Our results also revealed a significant correlation between increased levels of CRP in the serum and a decrease in ALM (*R* = −0.426 and *P* < 0.001; Figure 2(e)). Nevertheless, it is crucial to acknowledge that multiple investigations have been unsuccessful in establishing an association between elevated CRP levels and the deterioration of muscle mass. A meta-analysis was conducted on 19 separate cross-sectional studies involving 14,650 subjects, demonstrating that no publication bias in the correlation between muscle strength and either CRP or high-sensitivity C-reactive protein (hs-CRP) [30]. A different investigation involving 3,369 men aged 40–79 years examined the correlation between inflammatory markers and sarcopenia-related factors at the beginning of the observation period (2003–2005) and once more following a median follow-up duration of 4.29 years [31]. Moreover, they discovered no link between hs-CRP levels and reduced muscle mass. To summarize, the uncertain connection between heightened CRP levels and muscle mass is still not well-established. As a result, this study is the first to identify a noteworthy adverse causal link between CRP levels and ALM using the MR analysis method. A cohort study was carried out to examine the impact of serum CRP on the reduction of skeletal muscle mass in elderly women (67 ± 1.7 years) [32]. The study discovered that CRP has the ability to inhibit Akt phosphorylation and limit the activation of the subsequent mTORC1 pathway, leading to a decrease in muscle fibrin synthesis. Nevertheless, the exact mechanisms of how CRP negatively impacts skeletal muscle mass are not fully understood. More research is required to completely clarify the mechanisms by which CRP influences skeletal muscle and to examine potential therapeutic targets for alleviating its adverse effects.

The assessment of walking pace is crucial in the diagnosis of sarcopenia, serving as a practical and readily measurable indication of muscle functionality and overall physical performance [33]. A diminished walking pace often signals decreased muscle strength, as weaker muscles may not generate sufficient force for quicker and more efficient locomotion. Our investigation uncovered a noteworthy reverse association connecting the concentrations of CRP in the blood with 6-m walking speed (*R* = −0.431 and *P* < 0.001; Figure 2(f)), suggesting a deceleration in walking speed concurrent with increasing CRP levels. Our MR analysis further corroborated this observation, establishing a negative causal link between CRP levels and walking pace from a genetic standpoint. These findings imply that elevated CRP levels may not only be associated with reduced walking speed but could also contribute causally to this decline.

Furthermore, it is crucial to highlight the pathophysiological mechanisms that lead to sarcopenia, particularly prior to the overt loss of muscle function. Evidence increasingly suggests that systemic inflammation, mediated by inflammatory markers such as CRP, plays a pivotal role in the initiation and progression of muscle degradation and functional impairment [34]. Elevated CRP levels, indicative of increased inflammatory activity, are recognized not merely as a byproduct but as a facilitator of muscle catabolism [35]. This inflammatory environment is likely to exacerbate muscle protein breakdown and inhibit muscle regeneration, predisposing individuals to sarcopenia even before clinical symptoms appear. Additionally, our findings demonstrated an inverse relationship between CRP levels and muscle mass and strength, further supporting the utility of CRP as an early biomarker for sarcopenia. This relationship offers a window for early intervention, which could potentially reverse or slow the progression of muscle loss. By monitoring CRP levels, clinicians can identify individuals at risk early in the course of sarcopenia, enabling the timely implementation of therapeutic strategies aimed at reducing inflammation, thereby preserving muscle function and improving clinical outcomes in aging populations.

While our research shows potential, it is crucial to recognize the constraints of this study. First, the modest sample size in this cross-sectional study, confined to a single hospital, potentially affects the generalizability and statistical power of the results. Second, another limitation is the lack of data on genetic variants associated with sarcopenia in existing public GWAS database. This inadequacy arises due to the fact that the comprehension and identification of sarcopenia as per clinical consensus have materialized only in recent times. In spite of this limitation, our investigation employed the dependable indicators of sarcopenia, including grip strength, walking pace, and ALM, to furnish compelling proof endorsing an inverse cause-and-effect connection between CRP and sarcopenia. Third, the MR analysis used ALM data from the GWAS repository, acquired via BIA analysis. It is worth noting that BIA represents an indirect measurement technique and might possess lower precision in comparison to dual-energy X-ray absorptiometry (DXA), which presents an enhanced capability to evaluate body composition accurately and distinguish between different muscle clusters.

## 5. Conclusion

In conclusion, our findings demonstrate a robust association between the levels of CRP and the initiation of sarcopenia. The finding provides a potential mechanistic link between inflammaging and the decline in muscle mass observed in sarcopenia, highlighting the importance of addressing inflammation as a potential focus for intervention or management of sarcopenia.

## Figures and Tables

**Table 1:** Details of the GWAS summary statistics used in the research.

Category	Trait	Participants	Number of SNPs	Population	Datatype	GWAS ID
Exposure	C-reactive protein	436,939	4,231,728	European	Continuous	ebi-a-GCST90025959

Outcomes	Hand grip strength (left)	335,821	10,894,596	European	Continuous	ukb-a-374
	Hand grip strength (right)	335,842	10,894,596	European	Continuous	ukb-a-379
	Appendicular lean mass (ALM)	244,730	18,164,071	European	Continuous	ebi-a-GCST90000027
	Usual walking pace	335,349	10,894,596	European	Continuous	ukb-a-513

**Table 2:** Comparison of clinical variables between the sarcopenia and nonsarcopenia groups.

Clinical parameters	Sarcopenia (*n* = 74)	Nonsarcopenia (*n* = 133)	*P* value
^*∗*^Age (years)	75 (69–80)	59 (50–67)	**＜0.001**
^#^Sex			
Female	53 (71.62%)	56 (42.11%)	**＜0.001**
Male	21 (28.38%)	77 (57.89%)	
^#^BMI			
Normal weight	52 (70.27%)	78 (58.65%)	0.097
Abnormal weight	22 (29.73%)	55 (41.35%)	
^#^Anemic			
No	64 (86.49%)	131 (98.50%)	**＜0.001**
Yes	10 (13.51%)	2 (1.50%)	
^*∗*^Handgrip strength (kg)	14.4 (11.4–16.5)	32.2 (23.8–42.1)	**＜0.001**
^*∗*^ALM (kg/m^2^)	5.25 (5.11–5.84)	7.32 (6.14–7.98)	**＜0.001**
^※^6-m walk (m/s)	0.78 ± 0.19	1.39 ± 0.25	**＜0.001**
^*∗*^Calf circumference (cm)	30.4 (27.9–32.1)	34.1 (32.3–36.0)	**＜0.001**
^*∗*^Leukocyte count (10^9^/L)	6.33 (5.18–8.76)	7.06 (6.01–9.33)	**0.024**
^*∗*^Neutrophil count (10^9^/L)	4.52 (3.21–6.35)	4.41 (3.55–6.55)	0.294
^*∗*^CRP (mg/L)	8.94 (3.92–21.64)	1.55 (0.67–4.01)	**＜0.001**

^*∗*^indicates that the data are expressed as median (IQR) due to nonnormal distribution. ※ indicates that the data are expressed as mean ± SD due to normal distribution. #, the categorical parameters were shown as *n* (%). Statistically significant *P* values are indicated by bolded font.

**Table 3:** The results of univariate and multivariate binary logistics regression analysis.

Variable	Unadjusted			Adjusted ^*∗*^		
	OR	95% CI	*P* value	OR	95% CI	*P* value
CRP	1.152	1.094–1.214	**＜0.001**	1.151	1.070–1.238	**＜0.001**

^*∗*^indicates adjusted for age, sex, anemia, and leukocyte count. Bold values indicate *P*-values less than 0.05, which are considered statistically significant.

## Data Availability

The publicly accessible datasets for this research were obtained via the MR-Base platform. The corresponding author can provide the data utilized and analyzed in the cross-sectional inquiry upon a valid request.

## Supplementary Materials

Supplementary Materials Figure S1 presents forest plots of the causal effect of CRP on sarcopenia-related traits in MR analyses. Figure S2 shows the results of leave-one-out analyses for the MR analyses.
