# Supplementary material for: C-Reactive Protein Level as a Novel Serum Biomarker in Sarcopenia
Source: Mediators Inflamm. 2024 Aug 20;2024:3362336. doi: 10.1155/2024/3362336 (PMC11535261; doi:10.1155/2024/3362336)
Supplement: Supplementary Materials — Figure S1 presents forest plots of the causal effect of CRP on sarcopenia-related traits in MR analyses. Figure S2 shows the results of leave-one-out analyses for the MR analyses. [file 3362336.f1.docx]

Supplementary Figure:


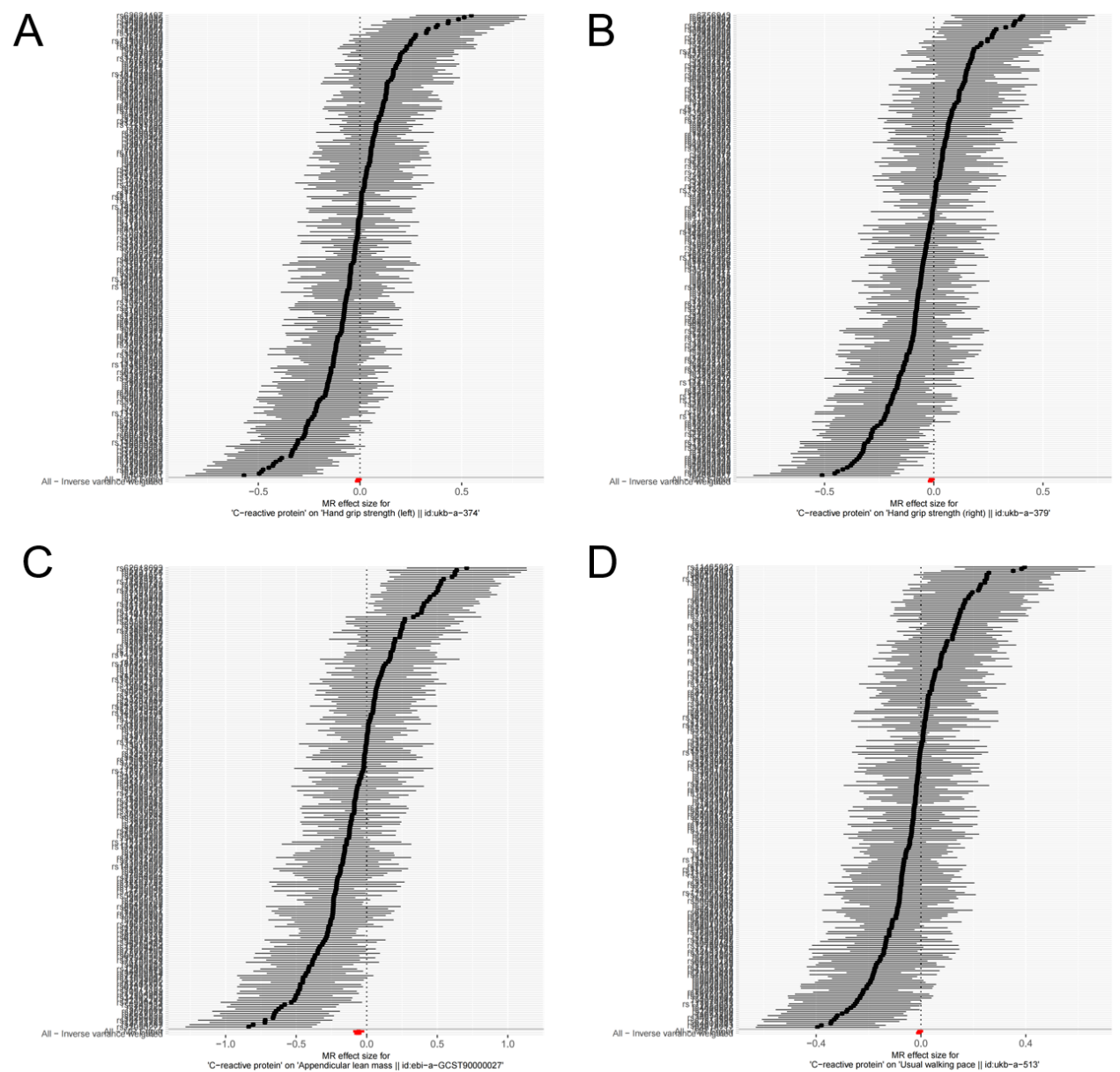


Supplementary Fig. 1. Forest plots of the causal effect of CRP on sarcopenia-related traits in MR analyses. (A) CRP-grip strength (left). (B) CRP-grip strength (right). (C) CRP-ALM. (D) CRP -Walking pace.


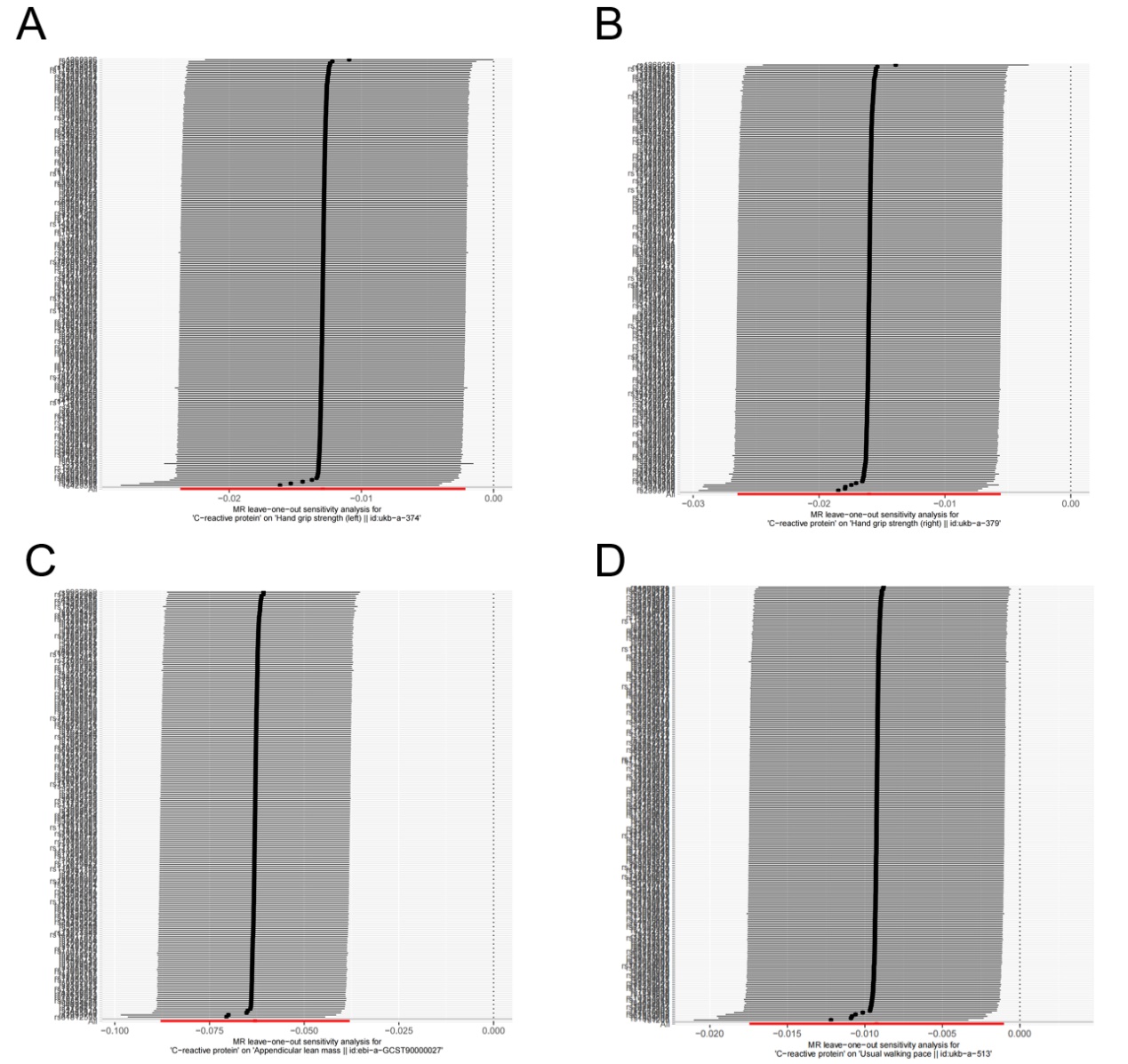


Supplementary Fig.2. Leave-one-out analysis. (A) CRP-grip strength (left). (B) CRP-grip strength (right). (C) CRP -ALM. (D) CRP -Walking pace.
